# Transactions of the American Surgical Association

**Published:** 1890-12

**Authors:** 


Transactions of the American Surgical Association. Volume the Eighth. Edited by J. Ewing Mears, M. D., Recorder of the Association. 8vo; pp. xx.—290. Philadelphia: Printed for the Association, and for sale by P. Blakiston, Son & Co. 1890. Dornan, Printer.

The deliberations of the American Surgical Association, contained in this volume, are quite up to the standard of former years. The transactions contain the following articles: President's address upon Pioneer Surgery in Kentucky, by David W. Yandell; Surgical Treatment of Tumors of the Bladder, by P. S. Conner; Treatment of Fractures of the Shaft of the Femur, by Stephen Smith; A Rare Form of Epithelioma of the Hand, by Charles B. Nancrede and H. Gibbes; The Pathology and Treatment of Clubfoot, especially varus and equino-varus, with brief reports of Fifteen Excisions of the Astragalus for Correction of the Deformity, by Thomas G. Morton; The Radical Cure of Hernia, with results of 134 operations, by W. T. Bull; Lumbar Hernia, by C. H. Mastin; The Propriety of the Removal of the Appendix Vermiformis during the Intervals of Recurrent Attacks of Appendicitis, by F. S. Dennis; Rupture of the Middle Meningeal Artery without Fracture; Ligature of the Common Carotid Artery for Secondary Hemorrhage, by Joseph Ransohoff; Nephrorrhaphy, by William Keen; Hypertrophy of the Prostate, by J. A. Cominger; Lupus of Tongue, by D. W. Cheever; Retro-Pharyngeal Sarcoma Removed by an External

Incision through the Neck, by D. W. Cheever; An Emergency Case, by J. E. Owens; A Report of Twenty-one Cases of Supra-Pubic Cystotomy, by Hunter McGuire; A New Method of Applying Antisepsis in the Treatment of Recent Anterior Urethritis, by J. William White; Arthrectomy of the Knee-Joint, by E. H. Bradford; Para-Nephritic Cysts, by Robert Abbe; Fractures and Disjunctures of the Pelvis: their Effect upon the Genito-Urinary Tract and Large Pelvic Blood-Vessels, by O. H. Allis; Removal of a Tubercular Tumor of the Larynx by Laryngo-Pharyngotomy, with Demonstration of Cases, by A. P. Gerster.

The selection of subjects is good, and the names of the writers are sufficient evidence that the contributions making up the transactions are masterly and scientific.

